# Supplementary material for: MetaRibo-Seq measures translation in microbiomes
Source: Nat Commun. 2020 Jun 29;11:3268. doi: 10.1038/s41467-020-17081-z (PMC7324362; doi:10.1038/s41467-020-17081-z)
Supplement: Supplementary file 10 — Supplementary Data 7 [file 41467_2020_17081_MOESM10_ESM.zip › File2/Confidence_High_Taxonomy/180847_out.krona.html]

Javascript must be enabled to view this page.

members
magnitude
magnitudeUnassigned
count
unassigned
taxon
rank

180847\_out

5

2
superkingdom
5

1239
5
phylum

186801
4
class

order
4
186802

2
species

SRS146812\_contig\_number\_34432SRS146813\_contig\_number\_21625
1950927

2
family
541000

genus
2
216851

species
2

SRS014613\_contig\_number\_17282SRS064276\_contig\_number\_35591
1946503


SRS019968\_contig\_number\_1492
species
1
1263000
